# Changes in the Suitable Habitats of Three Endemic Fishes to Climate Change in Tibet

**DOI:** 10.3390/biology11121808

**Published:** 2022-12-13

**Authors:** Tong Mu, Dekui He, Ren Zhu, Xiaoyun Sui, Yifeng Chen

**Affiliations:** 1 Laboratory of Biological Invasion and Adaptive Evolution, Institute of Hydrobiology, Chinese Academy of Sciences, Wuhan 430072, China; 2 University of Chinese Academy of Sciences, Beijing 100049, China

**Keywords:** climate change, species distribution model, endemic fish, suitable habitats, hydropower projects, Tibet

## Abstract

**Simple Summary:**

In this study, we predicted the suitable areas for three Tibetan fish in 2050 and 2090 under moderate and extreme climate change and explored the barrier effects of hydropower projects on the distribution areas of the three species. The three species are important to the local fishery and have received a wide range of attention. We found that they had obviously different responses under climate change, but they all had a tendency to move to higher areas. Moreover, our hydropower projects would hinder their migration. This study provides a reference for local fish protection in the future and a guideline for the scientific planning of hydropower development in Tibet.

**Abstract:**

As one of the most sensitive regions to global climate change, Tibet is subject to remarkable changes in biota over the past decades, including endemic fish species. However, no study has attempted to predict the changes in the distribution of Tibetan fishes, leaving a great blank for aquatic conservation in Tibet. Based on the Maximum Entropy model (MaxEnt), this study predicted the changes in the suitable habitats of three endemic fish species, including two species mainly inhabiting the rivers (*Glyptosternon maculatum*, *Oxygymnocypris stewartii*) and one species mainly inhabiting lakes (*Gymnocypris selincuoensis*) in Tibet under two representative concentration pathways (RCP2.6 and RCP8.5) under two future scenarios (2050 and 2090), and explored the impact of the barrier effects of hydropower projects on the suitable habitats of fish. The results showed that under the four scenarios, the net change in the suitable habitats of the *G. maculatum* was negative (−2.0–−18.8%), while the suitable habitats of the *O. stewartii* and *G. selincuoensis* would be expanded, with the net change of 60.0–238.3% and 46.4–56.9%, respectively. Under different scenarios, the suitable habitats of the three species had a tendency to migrate to a higher elevation, and the largest expansion in the range of migration was projected to occur under the 2090-RCP8.5 scenario. In addition, due to the impact of the hydropower projects, the ability of *G. maculatum* to obtain new suitable habitats from climate change would be reduced by 2.0–8.1%, which was less than the loss induced by climate change (5.5–25.1%), while the suitable habitats of *O. stewartii* would be reduced by 3.0–9.7%, which was more than the impact of climate change (about 1%). The results of this study have guiding significance for the conservation and management of fish resources diversity in the Yarlung Tsangpo River basin and Siling Co basin of Tibet, and also provide a reference for the coordination and scientific planning of hydropower projects in Tibet.

## 1. Introduction

As one of the most sensitive areas to global climate change [1,2], remarkable changes have been observed in the freshwater ecosystems in Tibet [3,4]. A large number of studies have proved that climate change will pose a profound impact on the biological properties, life histories and behaviors of fish [5,6,7,8], and Tao et al. [2] also revealed that climate change had different mechanisms on impacting river fishes and lake fishes. Changing distribution areas are one of the main responses of species to climate change among these changes brought about by climate change [9,10], and the affected population may show a decrease in the number of suitable habitats or migration to higher latitudes and elevations [5]. Freshwater fishes are considered to be more vulnerable to climate change, especially cold-water fish groups that are highly adapted to the extreme environment of the plateau with a limited capability of thermoregulation in their long-term evolution [11]. However, there have been no studies focusing on the changes to the suitable habitats of native fishes to climate change in Tibet.

The impact of climate change on fish diversity is usually accompanied by complex interactions between various human activities, which aggravate the impact [11,12,13]. In particular, the fragmentation of river networks caused by hydropower development may limit the migration capacity of fish and increase the risk of fish extinction [14,15]. Recent studies have confirmed that hydropower projects and other artificial obstacles will delay or prevent fish from migrating upstream with more suitable climate conditions due to the interaction together with climate change [16,17,18]. Herrera-R et al. [11] concerning Andean Amazon fish indicated that hydropower projects would have an effect on a large number of species when their suitable habitats contracted, reducing the potential biodiversity in some regions. These studies showed that it is necessary to take the physical barriers of fish migration into account when predicting the changes in the distribution range of freshwater fish induced by climate change [19].

Species distribution models (SDMs), establish the relationship between the distribution (or non-distribution) sample point information and environmental variables and apply this relationship to a model that estimates the distribution of target species in different environmental scenarios in other regions or in the future [20]. In recent years, species distribution models are widely used in different research areas, such as invasion biology, conservation biology, and biogeography [18,21,22,23,24,25,26]. Maximum Entropy model (MaxEnt) is open-source software that uses species occurrence records and environmental variables to build species distribution models [27,28]. This model can retain more useful information from the distribution data [29], with more predictive performance compared with other models [30,31,32].

*Glyptosternon maculatum* is known as the Sisoridae fish with the highest elevation of its distribution reaching 4200 m, which mainly feeds on benthic invertebrates. It is an endemic fish mainly inhabiting the Yarlung Tsangpo River, and a national second-class protected animal in China [33]. *Oxygymnocypris stewartii*, the only carnivorous fish in Tibet, only lives in the flowing water of the main trunk and tributaries in the upper and middle reaches of the Yarlung Tsangpo River. At the end of the last century, it has been listed in the Chinese Red species list, defined as an endangered species [34] and a national second-class protected animal [33], and was listed as a Near Threatened Species (NT) by IUCN [35]. *Gymnocypris selincuoensis*, an omnivorous fish, only distributes in the Siling Co basin in northern Tibet. When the breeding seasons come in, adults migrate to the tributaries, and juveniles migrate to the lake after being five years old [36]. It is a landmark fish in the North Tibetan National Park and dominates the fish community there.

In this study, three species of endemic fish, representing the flagship species of rivers and endorheic lakes, were selected for widespread attention in Tibet. Based on the species distribution model, we predicted the suitable habitats of the three species under current and different future scenarios and analyzed the impact of the barrier effect of hydropower project construction on the suitable habitats of the two river fishes in Yarlung Tsangpo River. Therefore, the aims of our study were to discuss the impact of climate change and hydropower projects towards suitable habitats of endemic fishes in Tibet and compare the different responses of river fish and lake fish, with a view to providing a scientific basis to assess the responses of endemic fish under future climate change in Tibet.

## 2. Materials and Methods

### 2.1. Climatic Variables

The data of elevation, slope, flow accumulation and flow length used for modeling were obtained from the data set of freshwater ecosystems with a resolution of 30 arc seconds in EarthEnv (http://www.earthenv.org/, accessed on 7 March 2022) [37]. The data of 19 bioclimatic predictors for the current climate of the years 1970–2000 were obtained from WorldClim 2.1 with a resolution of 30 arc seconds (http://www.worldclim.org/, accessed on 7 March 2022) [38]. In order to make relatively credible results, we cropped the environmental layers according to the natural distribution watershed of the three species and ran the model, respectively, for each species. In order to eliminate the influence of collinearity between environmental variables, we calculated the Pearson correlation coefficients between different variables to screen them [39]. When the absolute value of the correlation coefficient between variables was larger than 0.7, only the relative valuable factor among the significant correlation factors would be retained for modeling, evaluated by Jackknife analysis [40].

For the climate data under the future scenario, we selected the predicted values under two representative concentration pathways (RCP), moderate climate change (RCP2.6) and extreme climate change (RCP8.5) [18,41,42], provided by the Intergovernmental Panel on Climate Change Fifth Assessment Report to explore the response of three species of fish to climate change of different degrees in 2050 (the average value of 2041–2060) and 2090 (the average value of 2081–2100). Each concentration pathway was predicted using two global circulation models (ACCESS-ESM1-5 and FIO-ESM-2-0), which were superposed to take the average value, respectively. The ACCESS-ESM1-5 model was developed by the Commonwealth Scientific and Industrial Research Organization of Australia, which has been proven to be effective in simulating the temperature of the Qinghai–Tibet Plateau [43]. FIO-ESM-2-0 was developed by the First Institute of Oceanography, Ministry of Natural Resources of China, and its accuracy in reproducing climate fluctuations has been confirmed in previous studies [44,45].

### 2.2. Occurrence Records

**Figure 1:**
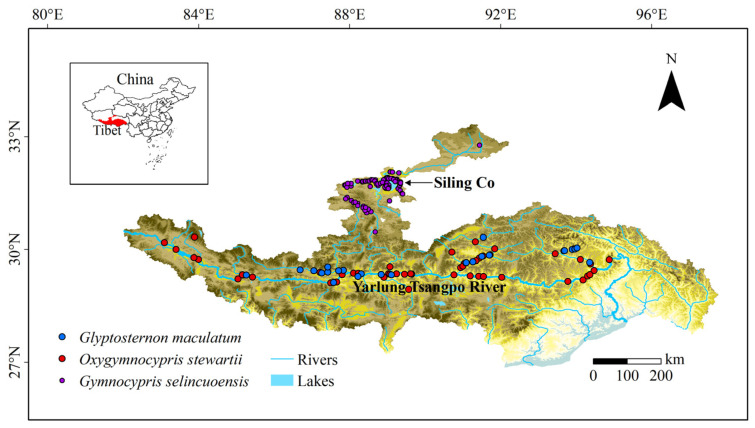
Species occurrences used for species distribution model.

Occurrence data of the three species were obtained from field surveys, published studies and online databases. In order to reduce the impact of sample record deviation on the prediction results, the Near tool in ArcGIS was used to capture the sample points outside the river network to the nearest river network. The longest capture distance was set as 1 km, and the sample points more than 1 km away from the river network were removed. Finally, 26 occurrences of *G. maculatum*, 57 occurrences of *O. stewartii* and 97 occurrences of *G. selincuoensis* were reserved for the prediction of the species distribution model (Figure 1).

### 2.3. Hydropower Projects Records

**Figure 2:**
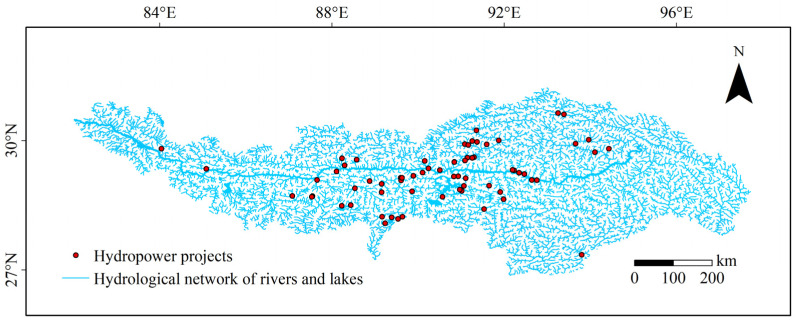
Distribution sketch map of the hydropower projects in Yarlung Tsangpo River basin.

A number of hydropower stations have been built and planned on the trunk and tributaries of the Yarlung Tsangpo River, while no hydropower station has been completed in the Siling Co basin. The locations of 80 hydropower projects in the Yarlung Tsangpo River basin (Figure 2) were obtained from published research [46] and the website of the management department. To reduce the impact of recording bias, the Near tool in ArcGIS was used to capture power projects located outside the river network to the nearest river network at a capture distance of 1 km. In this study, we mainly considered hydropower projects as a physical barrier to prevent fishes from changing the range of their suitable habitats induced by climate change, and the barrier effects of the power projects on the migration of fishes to the upstream and downstream were all considered. Based on the results under future climate change scenarios of the model, we removed the range of suitable areas to which species is unable to reach due to the barrier effects [47].

### 2.4. MaxEnt Modeling

The MaxEnt software was used to model the five scenarios of “Current”, “2050 moderate climate change (2050-RCP2.6)”, “2050 extreme climate change (2050-RCP8.5)”, “2090 young climate change (2090-RCP2.6)” and “2090 extreme climate change (2090-RCP8.5)”, respectively, to predict habitat suitability of the three species. In order to avoid overfitting the model and reduce the impact of sampling deviation, one occurrence was randomly selected from occurrences falling in the same grid cell [48]. In the process of model operation, the distribution data of each species were randomly divided, 25% of the data are used for testing, and 75% of the data were used for training. We chose “random seed” for each run, following the “subsample” way for 10 replications [49,50]. The maximum iterations were set to 5000 [51], and the jackknife method was used to measure the estimated relative contribution of each environmental variable.

### 2.5. Processing of Model Results

The quality of the model prediction was measured by the average of the area under the curve (AUC) of the receiver operating characteristic (ROC) curve [52]. In this study, only the model with good performance (AUC > 0.8) was retained [19], and the habitat suitability of each species was converted into presence or absence by using a threshold of maximizing the sum of sensitivity and specificity [53].

The changes in the suitable habitat distribution range of three species of fish were measured by four indicators, the gain of suitable habitats, the loss of suitable habitats, the net change of suitable habitats, and the average elevation of suitable habitats. Compared with the current time frame, the gain and loss of suitable habitats were the numbers of grid cells newly transformed into suitable and unsuitable habitats under the future scenarios [19]. The net change of suitable habitats was obtained by deducting the gain of suitable habitats from the loss of suitable habitats.

## 3. Results

### 3.1. Model Evaluation and Importance of Environmental Variables

The predictive performance of MaxEnt model for three species of fish reached a good or even excellent level (Figure S1). The AUC value of *G. maculatum* was 0.878, and the standard deviation was 0.064. The AUC value of *O. stewartii* was 0.926, with a standard deviation of 0.032. Moreover, the AUC value of *G. selincuoensis* was 0.807, and the standard deviation was 0.035.

After screening (Figure S2), different combinations of bioclimatic predictors were chosen for the three species (Table 1). Flow accumulation was the environmental factor that had the greatest contribution to the distribution of *G. maculatum* (81.4%). However, the river length had the highest contribution for the *O. stewartii* and *G. selincuoensis*, accounting for 66.3% and 66.5%, respectively.

### 3.2. Changes of Fish Habitats to Climate Change

**Figure 3:**
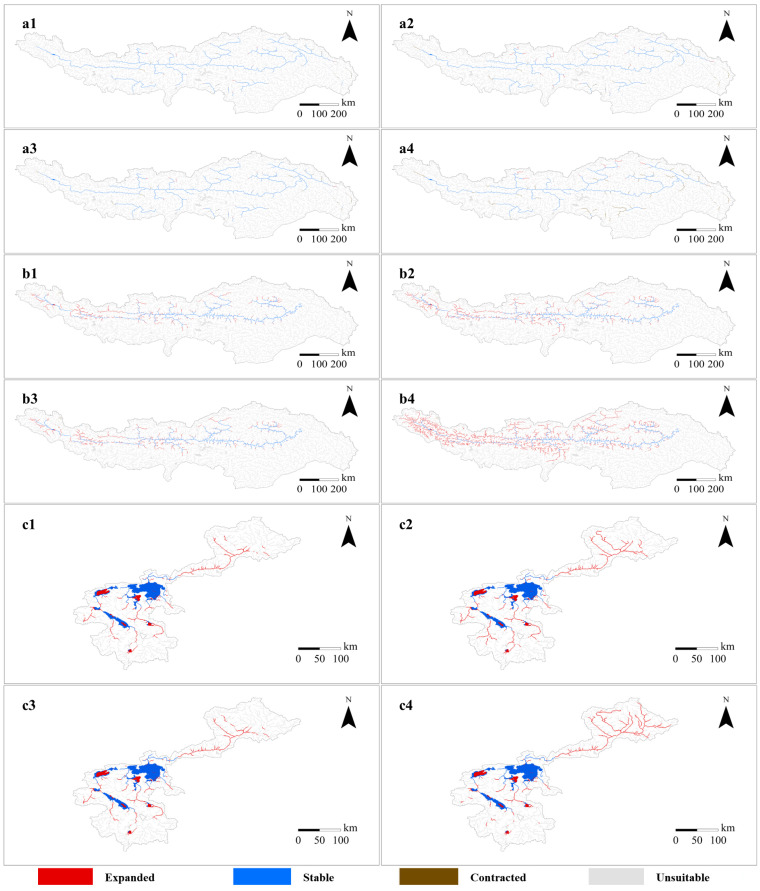
Changes in potential suitable habitats of three fish species under four scenarios. ((**a**): *Glyptosternon maculatum*; (**b**): *Oxygymnocypris stewartii*; (**c**): *Gymnocypris selincuoensis*; **1**: 2050-RCP2.6; **2**: 2050-RCP8.5; **3**: 2090-RCP2.6; **4**: 2090-RCP8.5).

The changes in suitable habitats of three fish species under four scenarios (2050-RCP2.6, 2090-RCP2.6, 2050-RCP8.5 and 2090-RCP8.5) were shown in Figure 3, and then we calculated the grid cells of suitable habitats of the three species under current and four future scenarios (Table 2). The three species demonstrated different trends of changes in suitable habitats. Under four future scenarios, the habitat net change for *G. maculatum* was negative (−2.0–−18.8%), while the net change for *O. stewartii* and *G. selincuoensis* was a positive number (60.0–238.3% and 46.4–56.9%, respectively). The common point of the three species was that the absolute value of habitat net change under RCP2.6 was lower than that under RCPP8.5 in both 2050 and 2090, and the value in 2050 was lower than that in 2090 under both RCP2.6 and RCP8.5. For *G. maculatum*, the loss of the currently suitable habitats exceeded the gain of suitable habitats in the four future scenarios. However, for both *O. stewartii* and *G. selincuoensis*, the loss of the suitable habitats was small (about 1.0%) or unchanged with a significant gain (60.9–239.1% and 46.4–56.9%, respectively) of suitable habitats. Obviously, the expansion extent of *O. stewartia* was more limited than *G. selincuoensis*. The changing trend of habitat gain for *O. stewartii* and *G. selincuoensis* was analogous to their net change, but for *G. maculatum*, the habitat gain did not reach its highest level under the 2090-RCP8.5 scenario compared with the other three scenarios.

### 3.3. Changes in Average Elevation of Fish Suitable Habitats to Climate Change

We calculated the average elevation of grid cells of the suitable habitats under each scenario (Table 3). Under different scenarios of future climate change, the average elevation of the three species all showed a trend of increase compared with the current scenarios, and the highest average elevation of suitable habitats for the three species all occurred in the 2090-RCP8.5 scenario. The average elevation of *G. maculatum* and *G. selincuoensis* under RCP8.5 were higher than that under RCP2.6 in both 2050 and 2090, and the average elevation in 2090 were higher than that in 2050 under both RCP2.6 and RCP8.5. The average elevation of *O. stewartii* under 2050-RCP2.6, 2050-RCP8.5 and 2090-RCP2.6 scenarios was similar.

### 3.4. Impact of River Fragmentation on Fish Caused by Hydropower Projects Construction

The results showed that the river fragmentation caused by hydropower development would have a barrier effect on the migration of fish. Compared with the current time frame, both the two river fishes, *G. maculatum* and *O. stewartii*, were both projected to reduce the extent of habitat gain obtained from climate change (Table 2). When we divided the cells’ amount of the reduced part by the amount in the current scenario, we obtained the percentage of the reduction. As for *G. maculatum*, the habitat gain would, respectively, be reduced by 2.0%, 8.1% and 2.4% under the scenarios of 2050-RCP2.6, 2050-RCP8.5 and 2090-RCP2.6, while the gain would not be affected by hydropower projects in 2090-RCP8.5 scenario. Moreover, for *O. stewartia*, the habitat gain would be reduced by 3.0%, 4.4%, 3.7% and 9.7%, respectively, under the 2050-RCP2.6, 2050-RCP8.5, 2090-RCP2.6 and 2090-RCP8.5 scenarios.

## 4. Discussion

### 4.1. Impact of Climate Change on the Range of Suitable Habitats of Three Species

Our study predicted the changes in the suitable habitats of three endemic fish species under four future scenarios of climate change and indicated the different responses in them. Under the four scenarios, the net change of the suitable habitats was negative for *G. maculatum*, while that of the *O. stewartii* and *G. selincuoensis* was positive. According to the research of Zhang [54], with a stronger swimming capacity and higher spindle shapes, the *O. stewartii* and *G. selincuoensis* of Cyprinidae may experience a greater expansion of their suitable habitats in the future and be more adaptive to the impact of climate change. Unlike the *O. stewartii* and *G. selincuoensis* of Cyprinidae, the *G. maculatum* of Sisoridae prefer to live under the rocks and between the crevices in torrential water [55], and they are not good at fast and long-distance swimming [55]. Perhaps limited by their distribution and migration capacity to gain more suitable habitats, they may be more sensitive to climate change, with a lower threshold of tolerance to climate change. Because of the limited adaptability to climate change, it may experience a greater loss of suitable habitats in the four future scenarios. Moreover, due to the significant decline of the *G. maculatum* population in recent years, the occurrence data we collected was relatively limited, which may lead to a lower threshold of its suitable habitats in the model.

In the four future scenarios, the growth trend and net change of suitable habitats of the *O. stewartii* and the *G. selincuoensis*, which are both Cyprinidae but distributed in different watersheds, were analogous, while their growth rate was different. That meant that there were some differences in their response degree to climate change. When the scenario changed with the extension of time and the concentration pathways from moderate to extreme, the trend in climate warming and humidifying was obvious in the basin, which would ease the restriction of the plateau extreme environment on organisms. Therefore, the two species of fish may be able to obtain more suitable habitats under the four scenarios. Referring to the environmental factors that affected the distribution of the two species, in the lake ecosystem, the rise of temperature can cause an increase in endogenous primary productivity, especially in the shallow water area of the lake shore. In the river ecosystem, low temperatures and low nutrient levels greatly limit the primary productivity of the river [56,57]. Moreover, aquatic organisms face great environmental pressure, which can produce obvious responses to weak environmental changes, not to mention that this region has been significantly affected by climate change [58]. In addition to the changes in temperature induced by climate change, surface runoff caused by increased rainfall will also bring more food resources to the river ecosystem. Compared with the lake ecosystem, the dynamic hydrological regime and physicochemical properties of river ecosystems will be more sensitive to climate change [59], while the body of water of lake ecosystems will be more stable. Therefore, under future scenarios, the *O. stewartii* in the Yarlung Tsangpo River basin may experience greater environmental changes than the *G. selincuoensis* in Lake Siling Co, and the growth in the number of suitable habitats may also be greater.

In addition, the specific response of species to climate change, such as the extent of contracting or expanding the range of suitable habitats, may largely depend on the life history characteristics related to the vulnerability and extinction risk of species [60]. This difference between river fish and lake fish in response to climate change needs to be further verified by similar research within the local scope, and this difference should also be paid enough attention to in the protection and management of the aquatic ecosystem in Tibet in the future, so as to better cope with climate change.

### 4.2. Impact of Climate Change on Migration of Suitable Habitats of Three Species

Consistent with other research results on the impact of climate change on freshwater fish, such as fish species in the Andean Amazon, Mekong River basin and 57% of species in the Lower Colorado River basin [11,54,61], our results showed that under four future scenarios, the average elevation of the suitable habitats was projected to be higher for both the three species and the highest average elevation were all occurred in the 2090-RCP8.5 scenario. Temperature is an important factor affecting the distribution of fish, and also the factor that contributes more to the model in this study (Table 1). In high-elevation areas, environments of low temperatures may limit the establishment and survival of fish populations. Under the background of global warming, the water temperature will also rise at the same time. Under the extreme climate change scenario in 2090, the average annual temperature of the Yarlung Tsangpo River basin and Siling Co basin would rise by 5.6–6.0 °C, which would greatly weaken the restrictions of the low-temperature environment on fish distribution [62]. That change may enhance the opportunity for fish populations to survive in colder areas and make their suitable habitats expand to higher elevation areas. The result showed that the migration tendency to respond to future climate change provides a reference for the protection of the three endemic species.

### 4.3. Impact of Hydropower Projects on Changes of Suitable Habitats of Three Species

The results of this study showed that for *G. maculatum*, the expansion of the suitable habitats under the scenarios of 2050-RCP2.6, 2050-RCP8.5 and 2090-RCP2.6 would be reduced by 2.0–8.1% caused by hydropower projects, which was less than the loss of its contemporary suitable habitats (5.5–25.1%) due to climate change. For *O. stewartii*, the habitat gain would also be reduced by 3.0–9.7% considering the impact of hydropower projects, which was greater than the loss of its contemporary suitable habitats induced by climate change (about 1.0%). Comparatively speaking, *G. maculatum* has stricter requirements for habitats, and their habitats are distributed in patches. They usually lay eggs in a river channel with a sand and gravel bottom [63], and their migration capacity is limited. Besides, the hydropower projects in the Yarlung Tsangpo River are mostly low-head dams, and the inundation area is relatively small compared with the whole basin. Therefore, compared with the impact of hydropower projects, the *G. maculatum* may be more sensitive to extreme climate change, resulting in a greater impact of climate change on the loss of its habitats. *O. stewartii* is a carnivorous fish that mainly feeds on fish. They have a stronger swimming capacity and a wider range of activities for hunting fish. Moreover, they also have different habitat preferences for wintering grounds and spawning grounds [64], which will cause a shift in their habitats. In addition, hydropower projects are often built at the main stream or larger tributaries to take a higher drop, which coincides with the habitats range of the *O. stewartii*. Therefore, it may be more susceptible to the barrier effects of hydropower projects. The different responses of the two fish species in the Yarlung Tsangpo River to climate change and hydropower projects should also be paid attention to for the protection of Tibetan endemic fishes in the future, so as to implement better protection measures according to the biological and ecological characteristics of different species.

However, the impact assessment of hydropower development in this study mainly focused on its impact on river connectivity, which played a role in physical barriers to fish migration. However, changes in flow, flow rate and flood pulse caused by hydropower development will also affect the foraging and spawning behavior of fish, and change the fish community structure [65]. We cannot quantify the impact of these factors on fish distribution at present, and more exhaustive research is needed. In addition, studies have shown that the changes in the upper and lower limits of suitable habitats may be determined by different mechanisms. The changes in the upper limit are related to the life history characteristics and nutritional environment of the species, while the changes in the lower limit are mainly related to thermal restriction [66]. Considering more complete characteristics related to reproduction, growth and nutrition mechanisms can better explain the response of species to climate change and hydropower projects [67].

## 5. Conclusions

This study quantified the ability to cope with climate change of three endemic fish species, providing a reference for the conservation and management of fish resources in the Yarlung Tsangpo River and Siling Co basins. At the same time, the quantitative results of the impacts of climate change and hydropower projects showed that the combined effects on fish will hinder the process of their adaptation to climate change, reduce their gain of new suitable habitats, and increase the risk of decline and extinction of populations, providing a basis for coordinating scientific planning of hydropower development and conversation of fish diversity.

## Figures and Tables

**Table 1:** Environmental variables used in MaxEnt with contribution and permutation importance.

Species	Environmental Variables	Contribution (%)	Permutation Importance
***Glyptosternon maculatum***	Flow accumulation	81.4	77.3
	Mean temperature of driest quarter (bio9)	10.1	9.1
	Precipitation of coldest quarter (bio19)	5.5	5.5
	Precipitation of wettest month(bio13)	1.4	7.3
	Slope	1.2	1.2
	Isothermality (bio3)	0.4	0.4
***Oxygymnocypris stewartii***	Flow length	66.3	32.6
	Max temperature of warmest month (bio5)	9.2	36.2
	Mean temperature of wettest quarter (bio8)	8.7	11.6
	Mean temperature of driest quarter (bio9)	7.5	12.8
	Slope	5.4	6.7
	Precipitation seasonality (bio15)	3	0.2
***Gymnocypris selincuoensis***	Flow length	66.5	34.3
	Isothermality (bio3)	12.7	21.9
	Max temperature of warmest month (bio5)	6.9	32.7
	Slope	6.8	6
	Temperature seasonality (bio4)	5.5	3.6
	Elevation	1.6	1.4

**Table 2:** Habitat changes of three species under four scenarios.

Scenarios	Glyptosternon Maculatum			Oxygymnocypris Stewartia			Gymnocypris Selincuoensis	
	Habitat Gain	Habitat Loss	Habitat Net Change	Habitat Gain	Habitat Loss	Habitat Net Change	Habitat Gain (Habitat Net Change)	Habitat Loss
2050-RCP2.6	204 (3.5%) ^1^	318 (5.5%)	−114 (2.0%)	2788 (60.9%)	41 (1.0%)	2747 (60.0%)	1778 (46.4%)	0
2050-RCP2.6-Dam	200	0	110	2705	0	2664	–	–
2050-RCP8.5	383 (6.6%)	628 (10.9%)	−245 (4.2%)	4446 (97.1%)	39 (1.0%)	4407 (96.2%)	2157 (56.3%)	0
2050-RCP8.5-Dam	352	0	214	4250	0	4211	–	–
2090-RCP2.6	206 (3.6%)	417 (7.2%)	−211 (3.7%)	3231 (70.6%)	32 (1.0%)	3199 (69.9%)	1819 (47.5%)	0
2090-RCP2.6-Dam	201	0	206	3112	0	3080	–	–
2090-RCP8.5	365 (6.3%)	1453 (25.1%)	−1088 (18.8%)	10949 (239.1%)	39 (1.0%)	10910 (238.3%)	2180 (56.9%)	0
2090-RCP8.5-Dam	365	0	−1088	9885	0	9846	–	–

^1^ The percentage of the habitat changes in the suitable habitats of current time frame.

**Table 3:** Average elevation of habitats of three species under four scenarios.

Scenarios	Average Elevation of Habitats (m)		
	*Glyptosternon* *maculatum*	*Oxygymnocypris* *stewartia*	*Gymnocypris* *selincuoensis*
Current	4891	4838	4800
2050-RCP2.6	4909	4874	4847
2050-RCP8.5	4928	4872	4863
2090-RCP2.6	4914	4867	4848
2090-RCP8.5	4948	4922	4873

## Data Availability

The data that support the findings of this study are available from the corresponding author upon reasonable request.

## Supplementary Materials

The following supporting information can be downloaded at: https://www.mdpi.com/article/10.3390/biology11121808/s1, Figure S1: Receiver operating characteristic (ROC) curve verification of distribution of *Glyptosternon maculatum* (a), *Oxygymnocypris stewartii* (b) and *Gymnocypris selincuoensis* (c) under different climate scenarios predicted by the Maxent model. Figure S2: Results of Jackknife tests of regularized training gain for the contribution of variables for *G. maculatum* (a), *O. stewartii* (b) and *G. selincuoensis* (c) under different climate scenarios predicted by the Maxent model.
