# Supplementary material for: Changes in the Suitable Habitats of Three Endemic Fishes to Climate Change in Tibet
Source: Biology (Basel). 2022 Dec 13;11(12):1808. doi: 10.3390/biology11121808 (PMC9774986; doi:10.3390/biology11121808)
Supplement: Supplementary file 1 [file biology-11-01808-s001.zip › Figure S1.pdf]

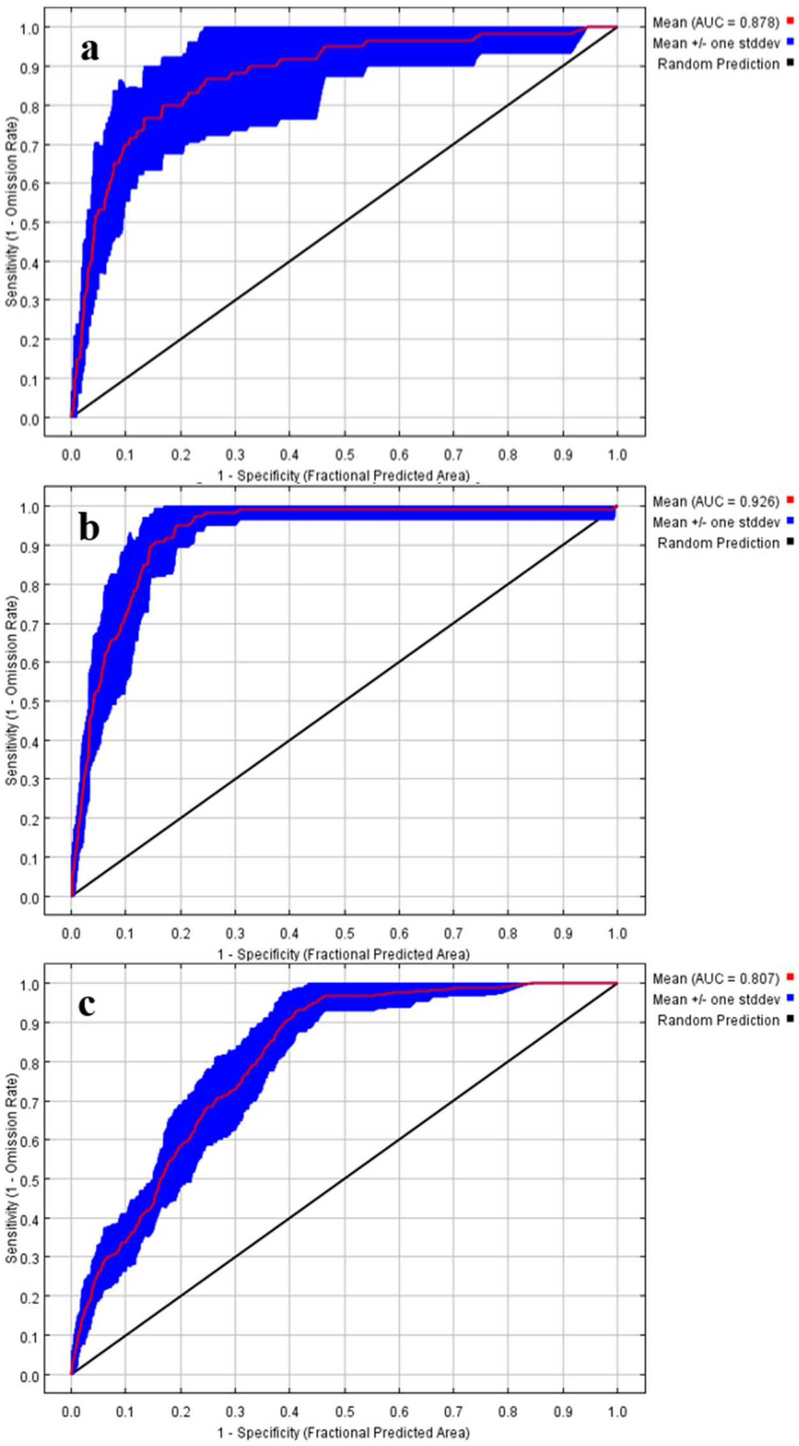

Figure S1. Receiver operating characteristic (ROC) curve verification of distribution of *Glyptosternon maculatum* (a), *Oxygymnocypris stewartii* (b) and *Gymnocypris selincuoensis* (c) under different climate scenarios predicted by the Maxent model.
