# Supplementary material for: Changes in the Suitable Habitats of Three Endemic Fishes to Climate Change in Tibet
Source: Biology (Basel). 2022 Dec 13;11(12):1808. doi: 10.3390/biology11121808 (PMC9774986; doi:10.3390/biology11121808)
Supplement: Supplementary file 1 [file biology-11-01808-s001.zip › Figure S2.pdf]

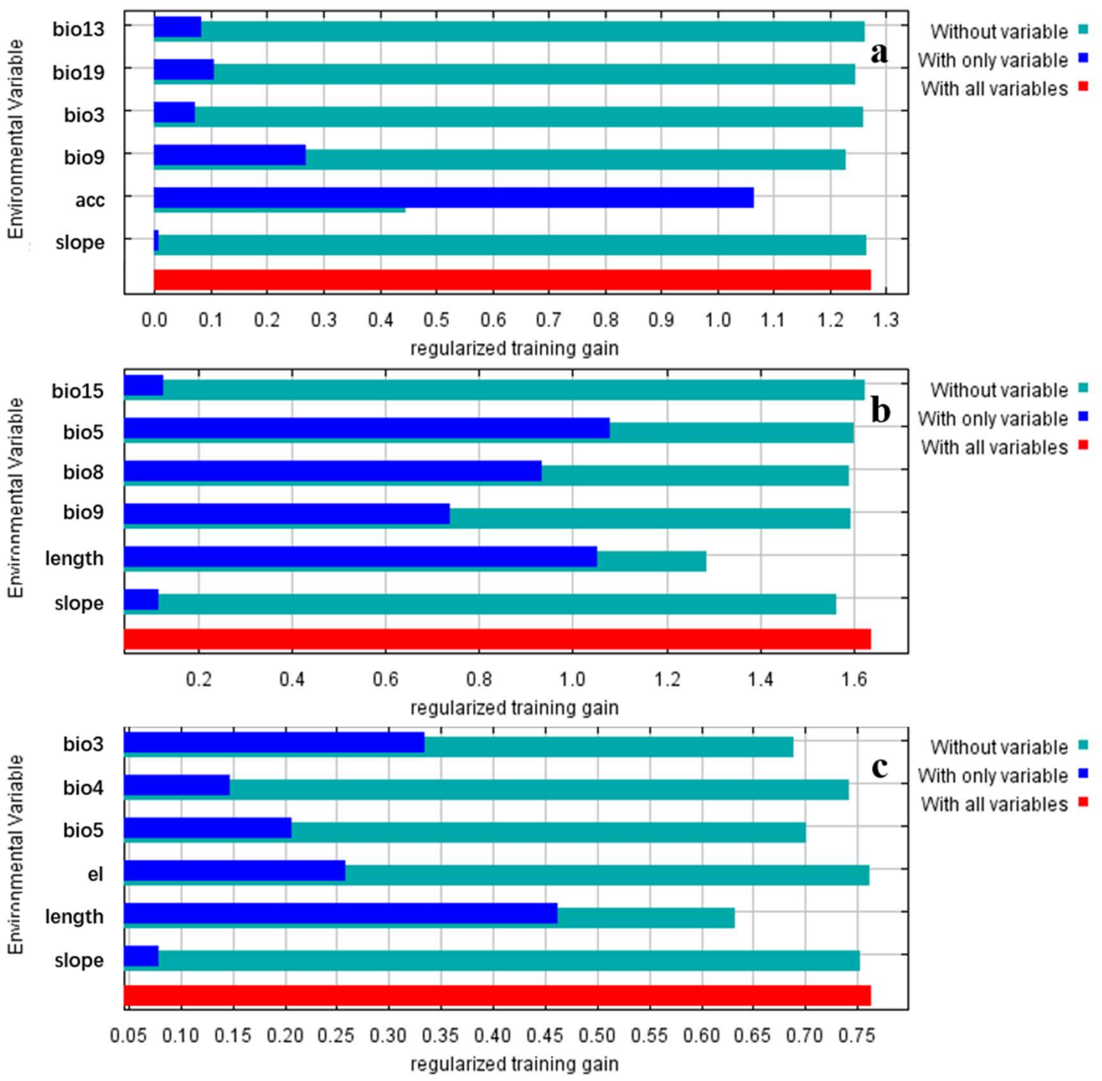

Figure S2. Results of Jackknife tests of regularized training gain for the contribution of variables for *G. maculatum* (a), *O. stewartii* (b) and *G. selincuoensis* (c) under different climate scenarios predicted by the Maxent model.
